# Supplementary material for: Geometric characterization of anomalous Landau levels of isolated flat bands
Source: Nat Commun. 2021 Nov 5;12:6433. doi: 10.1038/s41467-021-26765-z (PMC8571270; doi:10.1038/s41467-021-26765-z)
Supplement: Supplementary file 1 — Supplementary Information [file 41467_2021_26765_MOESM1_ESM.pdf]

Supplementary Information for "Geometric characterization of anomalous Landau levels of isolated flat bands"

Yoonseok Hwang,[1,2,3] Jun-Won Rhim,[1,2,4,*] and Bohm-Jung Yang[1,2,3,†]

[1]*Center for Correlated Electron Systems, Institute for Basic Science (IBS), Seoul 08826, Korea*
[2]*Department of Physics and Astronomy, Seoul National University, Seoul 08826, Korea*
[3]*Center for Theoretical Physics (CTP), Seoul National University, Seoul 08826, Korea*
[4]*Department of Physics, Ajou University, Suwon 16499, Korea*

Supplementary Note 1. Derivation of the relation between the Landau level spreading of an isolated flat band and cross-gap Berry connection

In the main text, the Landau level spreading of an isolated flat band is related to the cross-gap Berry connection and fidelity tensor through Eq. (5) of the main text. In this Supplementary Note, we derive Eq. (5) of the main text from the definition of the modified band structure $E_{n,B}(\mathbf{k})$ and the orbital magnetic moment $\mu_n(\mathbf{k})$, which are given by Eqs. (3) and (4) of the main text respectively. Recall that the modified band structure of nth band is expressed as

$$E_{n,B}(\mathbf{k}) = \varepsilon_n(\mathbf{k}) + \mu_n(\mathbf{k})B, \tag{1}$$

where $\varepsilon_n(\mathbf{k})$ denotes band dispersion of nth band in the zero magnetic field. The orbital magnetic moment $\mu_n(\mathbf{k})$ arises from the self rotation of wave packet corresponding to nth band, and it is given by

$$\mu_n(\mathbf{k}) = \frac{e}{\hbar}\,\mathrm{Im}\langle\partial_x u_n(\mathbf{k})|\left[\varepsilon_n(\mathbf{k}) - H(\mathbf{k})\right]|\partial_y u_n(\mathbf{k})\rangle, \tag{2}$$

where $\partial_i = \partial_{k_i}$ $(i = x, y)$ and $|u_n(\mathbf{k})\rangle$ denotes the periodic part of Bloch wave function of nth band. These results, Eqs. (1) and (2), were obtained by M.-C. Chang and Q. Niu [1].

Before deriving Eq. (5) of the main text, we review how Eqs. (3) and (4) of the main text can be obtained from the semiclassical theory of wave packet dynamics by closely following Refs. 1 and 2. First, let us consider a periodic Hamiltonian $H_0(\hat{\mathbf{r}},\hat{\mathbf{p}})$ and the Bloch wave function $|\psi_n(\mathbf{q})\rangle$ for nth band such that $H_0(\hat{\mathbf{r}},\hat{\mathbf{p}})|\psi_n(\mathbf{q})\rangle = \varepsilon_n(\mathbf{q})|\psi_n(\mathbf{q})\rangle$ where $\mathbf{q}$ is the crystal momentum. Correspondingly, the Bloch Hamiltonian, $H_{0\mathbf{q}}(\hat{\mathbf{r}},\hat{\mathbf{p}}) = e^{-i\mathbf{q}\cdot\hat{\mathbf{r}}}H_0(\hat{\mathbf{r}},\hat{\mathbf{p}})e^{i\mathbf{q}\cdot\hat{\mathbf{r}}}$, and the periodic part of Bloch wave function, $|u_n(\mathbf{q})\rangle = e^{i\mathbf{q}\cdot\hat{\mathbf{r}}}|\psi_n(\mathbf{q})\rangle$, satisfy a similar eigenvalue equation, $H_{0\mathbf{q}}(\hat{\mathbf{r}},\hat{\mathbf{p}})|u_n(\mathbf{q})\rangle = \varepsilon_n(\mathbf{q})|u_n(\mathbf{q})\rangle$. From now on, we omit the argument of Hamiltonian unless there is no confusion.

In the presence of constant magnetic field, the vector potential can be chosen as $\mathbf{A}(\mathbf{r}) = \frac{1}{2}\mathbf{B}\times\mathbf{r}$. As the vector potential changes the Hamiltonian according to the Peierls substitution, the Hamiltonian in the magnetic field becomes $H = H_0(\hat{\mathbf{r}},\hat{\mathbf{p}} + e\mathbf{A}(\hat{\mathbf{r}}))$. When a semiclassical wave packet is localized at $\mathbf{r}_c$ in real space, one can consider the derivative expansion of H near $\mathbf{r}_c$. Up to first order in B, the Hamiltonian is divided into *unperturbed* Hamiltonian H_1 and perturbation Hamiltonian H_2, i.e. $H = H_1 + H_2$. Explicitly, $H_{1,2}$ are expressed by

$$H_1 = H_0(\hat{\mathbf{r}},\hat{\mathbf{p}} + e\mathbf{A}(\mathbf{r}_c)), \quad H_2 = \frac{e}{4m}\mathbf{B}\cdot\left[(\hat{\mathbf{r}} - \mathbf{r}_c)\times\hat{\mathbf{P}}\right] + h.c, \tag{3}$$

where $\hat{\mathbf{P}} = -i\frac{m}{\hbar}[\hat{\mathbf{r}}, H_1]$ is the momentum operator. H_1 is the Hamiltonian that the wave packet experiences at its center $\mathbf{r}_c$. H_2 is the coupling between the orbital magnetic moment and the magnetic field. This can be seen by noticing that $\mathbf{L} = \frac{1}{2}(\hat{\mathbf{r}} - \mathbf{r}_c)\times\hat{\mathbf{P}} - \frac{1}{2}\hat{\mathbf{P}}\times(\hat{\mathbf{r}} - \mathbf{r}_c)$ where $\mathbf{L}$ is the angular momentum resulted from the self rotation of wave packet around its center. Hence, $H_2 = \frac{e}{2m}\mathbf{B}\cdot\mathbf{L} \simeq \mu\cdot\mathbf{B}$ where we define the orbital magnetic moment as $\mu \simeq \frac{e}{2m}\mathbf{L}$.

As we treat H_1 as the unperturbed Hamiltonian, the corresponding unperturbed eigenstates should be identified. Since $H_1 = H_0(\hat{\mathbf{r}},\hat{\mathbf{p}} + e\mathbf{A}(\mathbf{r}_c)) = e^{-ie\mathbf{A}(\mathbf{r}_c)\cdot\hat{\mathbf{r}}}H_0(\hat{\mathbf{r}},\hat{\mathbf{p}})e^{ie\mathbf{A}(\mathbf{r}_c)\cdot\hat{\mathbf{r}}}$, one can define $H_{1\mathbf{q}} = e^{-i\mathbf{q}\cdot\hat{\mathbf{r}}}H_1e^{i\mathbf{q}\cdot\hat{\mathbf{r}}} = e^{-i\mathbf{k}\cdot\hat{\mathbf{r}}}H_0e^{i\mathbf{k}\cdot\hat{\mathbf{r}}}$ by introducing the gauge-invariant crystal momentum $\mathbf{k} = \mathbf{q} + \frac{e}{\hbar}\mathbf{A}(\mathbf{r}_c)$. From $H_{1\mathbf{q}} = e^{-i\mathbf{k}\cdot\hat{\mathbf{r}}}H_0e^{i\mathbf{k}\cdot\hat{\mathbf{r}}}$, $H_{1\mathbf{q}}$ can also be identified as $H_{0\mathbf{k}}$. Hence, the Bloch wave function and its periodic part must be chosen such that $H_{0\mathbf{k}}|u_n(\mathbf{k})\rangle = \varepsilon_n(\mathbf{k})|u_n(\mathbf{k})\rangle$, $H_1|\psi_n(\mathbf{k})\rangle = \varepsilon_n(\mathbf{k})|\psi_n(\mathbf{k})\rangle$, and $|\psi_n(\mathbf{k})\rangle = e^{i\mathbf{q}\cdot\hat{\mathbf{r}}}|u_n(\mathbf{k})\rangle$. Once the unperturbed eigenstates are identified, a semiclassical wave packet $|W_n\rangle$ can be constructed as follows. $|W_n\rangle$ is a superposition of $|\psi_n(\mathbf{k})\rangle$ with different momenta $\mathbf{k}$: $|W_n\rangle = \sum_{\mathbf{q}} w(\mathbf{q})|\psi_n(\mathbf{k})\rangle = \sum_{\mathbf{q}} w(\mathbf{q})e^{i\mathbf{q}\cdot\hat{\mathbf{r}}}|u_n(\mathbf{k})\rangle$ where the amplitude $w(\mathbf{q})$ are normalized according to $\sum_{\mathbf{q}} \overline{w(\mathbf{q})}w(\mathbf{q}) = 1$. Since the wave packet

* phyruth@gmail.com
† bjyang@snu.ac.kr

is localized at real-space center $\mathbf{r}_c$ and momentum-space center $\mathbf{q}_c$, the amplitude $w(\mathbf{q})$ must satisfy $\langle W_n|\hat{\mathbf{r}}|W_n\rangle = \mathbf{r}_c$ and $\overline{w(\mathbf{q})}w(\mathbf{q}) \simeq \delta_{\mathbf{q},\mathbf{q}_c}$. Note that $\overline{x}$ and δ denote the complex conjugation of x and the Kronecker delta symbol, respectively.

Now, we can calculate the energy expectation value $\langle W_n|H|W_n\rangle = E_1 + E_2$ where $E_{1,2} = \langle W_n|H_{1,2}|W_n\rangle$. First, E_1 can be obtained straightforwardly,

$$E_1 = \langle W_n|H_1|W_n\rangle = \sum_{\mathbf{q}} \overline{w(\mathbf{q})}w(\mathbf{q})\varepsilon_n(\mathbf{k}) \simeq \varepsilon_n(\mathbf{k}_c), \tag{4}$$

where $\mathbf{k}_c = \mathbf{q}_c + \frac{e}{\hbar}\mathbf{A}(\mathbf{r}_c)$. Hence, E_1 is nothing but the unperturbed band dispersion at momentum $\mathbf{k}_c$. The energy correction E_2 can be written as

$$\begin{aligned}
\langle W_n|H_2|W_n\rangle &= \sum_{\mathbf{q},\mathbf{q}'} \overline{w(\mathbf{q}')}w(\mathbf{q})\langle\psi_n(\mathbf{k}')|H_2|\psi_n(\mathbf{k})\rangle \\
&= -i\frac{e}{4\hbar}\mathbf{B}\cdot\sum_{\mathbf{q},\mathbf{q}'} \overline{w(\mathbf{q}')}w(\mathbf{q})\langle u_n(\mathbf{k}')|(\hat{\mathbf{r}}-\mathbf{r}_c)\times e^{-i\mathbf{q}'\cdot\hat{\mathbf{r}}}[\hat{\mathbf{r}},H_1]e^{i\mathbf{q}\cdot\hat{\mathbf{r}}}|u_n(\mathbf{k})\rangle + h.c. \\
&= \frac{e}{4\hbar}\mathbf{B}\cdot\sum_{\mathbf{q},\mathbf{q}'} \overline{w(\mathbf{q}')}w(\mathbf{q})\langle u_n(\mathbf{k}')|(\hat{\mathbf{r}}-\mathbf{r}_c)e^{-i(\mathbf{q}'-\mathbf{q})\cdot\hat{\mathbf{r}}}\times\partial_{\mathbf{k}}H_{0\mathbf{k}}|u_n(\mathbf{k})\rangle + h.c., \\
&= \frac{e}{4\hbar}\mathbf{B}\cdot\sum_{\mathbf{q},\mathbf{q}'} \overline{w(\mathbf{q}')}w(\mathbf{q})\mathbf{F}(\mathbf{k}',\mathbf{k}) + h.c.,
\end{aligned} \tag{5}$$

where $[\hat{\mathbf{r}}, H_{0\mathbf{k}}] = i\partial_{\mathbf{k}}H_{0\mathbf{k}}$ is applied in the third equality and we define $\mathbf{F}(\mathbf{k}',\mathbf{k}) = \langle u_n(\mathbf{k}')|(\hat{\mathbf{r}}-\mathbf{r}_c)e^{-i(\mathbf{q}'-\mathbf{q})\cdot\hat{\mathbf{r}}}\times\partial_{\mathbf{k}}H_{0\mathbf{k}}|u_n(\mathbf{k})\rangle$ for notational simplicity. The expression for $F(\mathbf{k}',\mathbf{k})$ can be simplified with the help of various identities such as $\hat{\mathbf{r}} = i\partial_{\mathbf{q}}e^{-i\mathbf{q}\cdot\hat{\mathbf{r}}}e^{i\mathbf{q}\cdot\hat{\mathbf{r}}}$ and $\partial_{\mathbf{k}}H_{0\mathbf{k}}|u_n(\mathbf{k})\rangle = \partial_{\mathbf{k}}\varepsilon_n(\mathbf{k})|u_n(\mathbf{k})\rangle + (\varepsilon_n(\mathbf{k}) - H_{0\mathbf{k}})|\partial_{\mathbf{k}}u_n(\mathbf{k})\rangle$. We also use $\langle f_1(\mathbf{q}')|e^{-i(\mathbf{q}'-\mathbf{q})\cdot\hat{\mathbf{r}}}|f_2(\mathbf{q})\rangle = \langle f_1(\mathbf{q})|f_2(\mathbf{q})\rangle\delta_{\mathbf{q}',\mathbf{q}}$, which holds for two states $|f_{1,2}(\mathbf{q})\rangle$ that are periodic in real space. Applying these identities, we obtain

$$\begin{aligned}
\mathbf{F}(\mathbf{k}',\mathbf{k}) &= i\langle u_n(\mathbf{k}')|\left[(\partial_{\mathbf{q}'} - \mathbf{r}_c)e^{-i(\mathbf{q}'-\mathbf{q})\cdot\hat{r}}\right]\times\left[\partial_{\mathbf{k}}\varepsilon_n(\mathbf{k})|u_n(\mathbf{k})\rangle + (\varepsilon_n(\mathbf{k}) - H_{0\mathbf{k}})|\partial_{\mathbf{k}}u_n(\mathbf{k})\rangle\right] \\
&= (i\partial_{\mathbf{k}'}\delta_{\mathbf{k}',\mathbf{k}} - \mathbf{r}_c + A_n(\mathbf{k}))\times\partial_{\mathbf{k}}\varepsilon_n(\mathbf{k}) - i\langle\partial_{\mathbf{k}}u_n(\mathbf{k})|\times\left[\varepsilon_n(\mathbf{k}) - H_{0\mathbf{k}}\right]|\partial_{\mathbf{k}}u_n(\mathbf{k})\rangle,
\end{aligned} \tag{6}$$

where $A_n(\mathbf{k}) = -i\langle\partial_{\mathbf{k}}u_n(\mathbf{k})|u_n(\mathbf{k})\rangle$. Here, we treat the Kronecker delta as the Dirac delta function with the appropriate numerical factor. The substitution of Eq. (6) into Eq. (5) leads to

$$\begin{aligned}
E_2 =& -\frac{ie}{4\hbar}\mathbf{B}\cdot\sum_{\mathbf{q}}|w(\mathbf{q})|^2\langle\partial_{\mathbf{k}}u_n(\mathbf{k})|\times\left[\varepsilon_n(\mathbf{k}) - H_{0\mathbf{k}}\right]|\partial_{\mathbf{k}}u_n(\mathbf{k})\rangle \\
&+ \frac{e}{4\hbar}\mathbf{B}\cdot\sum_{\mathbf{q}}\left[-iw(\mathbf{q})\partial_{\mathbf{q}}\overline{w(\mathbf{q})} + (A_n(\mathbf{k}) - \mathbf{r}_c)|w(\mathbf{q})|^2\right]\times\partial_{\mathbf{k}}\varepsilon_n(\mathbf{k}) + h.c. \\
\simeq& -\frac{ie}{4\hbar}\mathbf{B}\cdot\langle\partial_{\mathbf{k}_c}u_n(\mathbf{k}_c)|\times\left[\varepsilon_n(\mathbf{k}_c) - H_{0\mathbf{k}_c}\right]|\partial_{\mathbf{k}_c}u_n(\mathbf{k}_c)\rangle \\
&+ \frac{e}{4\hbar}\mathbf{B}\cdot\left[-iw(\mathbf{q}_c)\partial_{\mathbf{q}_c}\overline{w(\mathbf{q}_c)} + (A_n(\mathbf{k}_c) - \mathbf{r}_c)|w(\mathbf{q}_c)|^2\right]\times\partial_{\mathbf{k}_c}\varepsilon_n(\mathbf{k}_c) + h.c.
\end{aligned} \tag{7}$$

As the condition, $\langle W_n|\hat{\mathbf{r}}|W_n\rangle = \mathbf{r}_c$, imposes $iw(\mathbf{q}_c)\partial_{\mathbf{q}_c}\overline{w(\mathbf{q}_c)} = (A_n(\mathbf{k}_c) - \mathbf{r}_c)|w(\mathbf{q}_c)|^2$, we finally obtain

$$E_2 = -\frac{ie}{4\hbar}\mathbf{B}\cdot\langle\partial_{\mathbf{k}_c}u_n(\mathbf{k}_c)|\times\left[\varepsilon_n(\mathbf{k}_c) - H_{0\mathbf{k}_c}\right]|\partial_{\mathbf{k}_c}u_n(\mathbf{k}_c)\rangle + h.c.. \tag{8}$$

Since this result holds for any wave packet with $\mathbf{k}_c$, $\mathbf{k}_c$ can be replaced with $\mathbf{k}$. Hence, $E_2 = \boldsymbol{\mu}(\mathbf{k})\cdot\mathbf{B}$ where the orbital magnetic moment is defined by

$$\boldsymbol{\mu}(\mathbf{k}) = -\frac{ie}{4\hbar}\langle\partial_{\mathbf{k}}u_n(\mathbf{k})|\times\left[\varepsilon_n(\mathbf{k}) - H_{0\mathbf{k}}\right]|\partial_{\mathbf{k}}u_n(\mathbf{k})\rangle + h.c., \tag{9}$$

The z component of $\boldsymbol{\mu}(\mathbf{k})$ is identical to Eq. (2).

Equipped with the above result, we now derive Eq. (5) of the main text from the orbital magnetic moment formula in Eq. (2). While we are interested in the Landau level spreading of the flat band, let us assume that the flat band is the n-th band with

energy ε_0. Then, Eq. (2) becomes

$$\mu_n(\mathbf{k}) = \frac{e}{\hbar}\,\mathrm{Im}\langle\partial_x u_n(\mathbf{k})|\,[\varepsilon_0 - H(\mathbf{k})]\,|\partial_y u_n(\mathbf{k})\rangle, \tag{10}$$

$$= \frac{e}{\hbar}\,\mathrm{Im}\langle\partial_x u_n(\mathbf{k})|\sum_l |u_l(\mathbf{k})\rangle\langle u_l(\mathbf{k})|\,[\varepsilon_0 - H(\mathbf{k})]\sum_m |u_m(\mathbf{k})\rangle\langle u_m(\mathbf{k})|\partial_y u_n(\mathbf{k})\rangle, \tag{11}$$

$$= \frac{e}{\hbar}\,\mathrm{Im}\sum_{l,m}[\varepsilon_0 - \varepsilon_m(\mathbf{k})]\,\langle\partial_x u_n(\mathbf{k})|u_l(\mathbf{k})\rangle\langle u_m(\mathbf{k})|\partial_y u_n(\mathbf{k})\rangle\delta_{lm}, \tag{12}$$

$$= \frac{e}{\hbar}\,\mathrm{Im}\sum_{m}[\varepsilon_0 - \varepsilon_m(\mathbf{k})]\,\langle\partial_x u_n(\mathbf{k})|u_m(\mathbf{k})\rangle\langle u_m(\mathbf{k})|\partial_y u_n(\mathbf{k})\rangle, \tag{13}$$

where the completeness relation $I = \sum_m |u_m(\mathbf{k})\rangle\langle u_m(\mathbf{k})|$ is used. Without loss of generality, one can assume that $\varepsilon_0 = 0$. Then, by inserting Eq. (13) into the Eq. (1), we obtain

$$E_{n,B}(\mathbf{k}) = \varepsilon_n(\mathbf{k}) + \mu_n(\mathbf{k})B = \mu_n(\mathbf{k})B, \tag{14}$$

$$= -B\frac{e}{\hbar}\,\mathrm{Im}\sum_m \varepsilon_m(\mathbf{k})\langle\partial_x u_n(\mathbf{k})|u_m(\mathbf{k})\rangle\langle u_m(\mathbf{k})|\partial_y u_n(\mathbf{k})\rangle, \tag{15}$$

$$= -2\pi\frac{\phi}{\phi_0}\,\mathrm{Im}\sum_m \varepsilon_m(\mathbf{k})\langle\partial_x u_n(\mathbf{k})|u_m(\mathbf{k})\rangle\langle u_m(\mathbf{k})|\partial_y u_n(\mathbf{k})\rangle, \tag{16}$$

where A_0 is the area of the unit cell, $\phi = BA_0$, and $\phi_0 = h/e$. Finally, noting that the fidelity tensor is defined by

$$\chi_{ij}^{nm}(\mathbf{k}) = \langle\partial_i u_n(\mathbf{k})|u_m(\mathbf{k})\rangle\langle u_m(\mathbf{k})|\partial_j u_n(\mathbf{k})\rangle = A_i^{nm}(\mathbf{k})^* A_j^{nm}(\mathbf{k}), \tag{17}$$

we obtain

$$E_{n,B}(\mathbf{k}) = -2\pi\frac{\phi}{\phi_0}\frac{1}{A_0}\mathrm{Im}\sum_{m\neq n}\varepsilon_m(\mathbf{k})\chi_{xy}^{nm}(\mathbf{k}). \tag{18}$$

Since we assume that the flat band's energy is zero, one should interpret $\varepsilon_m(\mathbf{k})$ in Eq. (18) as the energy of the m-th band with respect to the flat band energy.

Supplementary Note 2. Symmetry constraint on the Landau level spreading of flat band

In this Supplementary Note, we study the symmetry transformation of the modified band dispersion $E_{n,B}(\mathbf{k})$ and the fidelity tensor $\chi_{ij}^{nm}(\mathbf{k})$. Here, we use a compact notation which can be applied to both unitary and anti-unitary (anti-)symmetries. In this notation, any symmetry operation $\hat{\sigma}$ can be expressed by

$$U_\sigma(\mathbf{k})\overline{H(\mathbf{k})}^s U_\sigma(\mathbf{k})^\dagger = pH(O_\sigma\mathbf{k}), \tag{19}$$

$$\mathfrak{g}_\sigma = (O_\sigma, s, p), \tag{20}$$

where $s = 0, 1$ and $p = \pm 1$. The bar notation denotes the complex conjugation, i.e., $\overline{x} = x^*$. For example, $\mathfrak{g}_\sigma = (\mathbb{1}, 0, -1)$ and $(-\mathbb{1}, 1, 1)$ correspond to chiral and time-reversal symmetries, respectively, where $\mathbb{1}$ denotes the identity matrix. First consequence of symmetry in Eq. (20) is that the band structure is symmetric with respect to σ. Namely, $p\,\varepsilon(\mathbf{k})$ is the corresponding energy eigenvalue at $O_\sigma\mathbf{k}$ for an energy eigenvalue $\varepsilon(\mathbf{k})$ in the band structure. Furthermore, symmetry relations are imposed on the energy eigenstates as follows.

$$U_\sigma(\mathbf{k})\overline{|u_m(\mathbf{k})\rangle}^s = |u_{m_\sigma}(O_\sigma\mathbf{k})\rangle B_\sigma(\mathbf{k})_{m_\sigma m} \quad (m \neq n), \tag{21}$$

$$U_\sigma(\mathbf{k})\overline{|u_n(\mathbf{k})\rangle}^s = |u_n(O_\sigma\mathbf{k})\rangle e^{i\phi_n(\mathbf{k})}, \tag{22}$$

for dispersive bands m ($m \neq n$) and the flat band n, respectively. Here, the band indices are defined such that $\varepsilon_m(\mathbf{k}) = p\,\varepsilon_{m_\sigma}(O_\sigma\mathbf{k})$, and $B_\sigma(\mathbf{k})_{m_\sigma m}$ and $e^{i\phi_n(\mathbf{k})}$ are unitary and periodic in the Brillouin zone. With the symmetry transformation of energy eigenstates defined above in Eqs. (21) and (22), one can derive symmetry constraint on the modified band dispersion $E_{n,B}(\mathbf{k})$ and the fidelity tensor $\chi_{ij}^{nm}(\mathbf{k})$.

Symmetry constraint on the modified band dispersion $E_{n,B}(\mathbf{k})$

We recall the definition of modified band dispersion $E_{n,B}(\mathbf{k})$:

$$E_{n,B}(\mathbf{k}) = -2\pi\frac{\phi}{\phi_0}\frac{1}{A_0}\mathrm{Im}\langle\partial_x u_n(\mathbf{k})|H(\mathbf{k})|\partial_y u_n(\mathbf{k})\rangle. \tag{23}$$

Note that n-th band is an isolated flat band with $\varepsilon_n(\mathbf{k}) = 0$. Now, we study the symmetry transformation of $l^n_{ij}(\mathbf{k}) := \langle\partial_i u_n(\mathbf{k})|H(\mathbf{k})|\partial_j u_n(\mathbf{k})\rangle$ by using Eq. (22):

$$\begin{aligned} l^n_{ij}(O_\sigma\mathbf{k}) =& \left[\langle\partial_{\mathbf{k}'_i} u_n(\mathbf{k}')|H(\mathbf{k}')|\partial_{\mathbf{k}'_j} u_n(\mathbf{k}')\rangle\right]_{\mathbf{k}'=O_\sigma\mathbf{k}} \\ =&[O_\sigma]_{ii'}[O_\sigma]_{jj'}\,\partial_{i'}\left(e^{i\phi_n(\mathbf{k})}\overline{\langle u_n(\mathbf{k})|}^s U_\sigma(\mathbf{k})^\dagger\right) \\ &\times\left(pU_\sigma(\mathbf{k})\overline{H(\mathbf{k})}^s U_\sigma(\mathbf{k})^\dagger\right)\,\partial_{j'}\left(e^{-i\phi_n(\mathbf{k})}U_\sigma(\mathbf{k})\overline{|u_n(\mathbf{k})\rangle}^s\right) \\ =&p[O_\sigma]_{ii'}[O_\sigma]_{jj'}\,\overline{\langle\partial_{i'} u_n(\mathbf{k})|H(\mathbf{k})|\partial_{j'} u_n(\mathbf{k})\rangle}^s \\ =&p[O_\sigma]_{ii'}[O_\sigma]_{jj'}\,\overline{l^n_{i'j'}(\mathbf{k})}^s. \end{aligned} \tag{24}$$

Hence, we obtain

$$\begin{aligned} E_{n,B}(O_\sigma\mathbf{k}) =& -2\pi\frac{\phi}{\phi_0}\frac{1}{A_0}\,\mathrm{Im}\left[p\,[O_\sigma]_{xi}[O_\sigma]_{yj}\,\overline{l^n_{ij}(\mathbf{k})}^s\right] \\ =& -(-1)^s p\,\left([O_\sigma]_{xx}[O_\sigma]_{yy} - [O_\sigma]_{xy}[O_\sigma]_{yx}\right)\times 2\pi\frac{\phi}{\phi_0}\frac{1}{A_0}\,\mathrm{Im}\,l^n_{xy}(\mathbf{k}) \\ =&(-1)^s p\,\mathrm{Det}O_\sigma\,E_{n,B}(\mathbf{k}). \end{aligned} \tag{25}$$

Note that $l^n_{xx}(\mathbf{k})$ and $l^n_{yy}(\mathbf{k})$ are real, thus $\mathrm{Im}\,l^n_{xx}(\mathbf{k}) = \mathrm{Im}\,l^n_{yy}(\mathbf{k}) = 0$. We remark two cases when the LLS is strongly constrained by symmetry. First, when $(-1)^s p = -1$ and $O_\sigma = \mathbb{1}$ are satisfied at the same time, the modified band dispersion $E_{n,B}(\mathbf{k}) = 0$ vanishes. Chiral symmetry C and space-time-inversion symmetry I_{ST}, characterized by $\mathfrak{g}_C = (\mathbb{1}, 0, -1)$ and $\mathfrak{g}_{I_{\mathrm{ST}}} = (\mathbb{1}, 1, 1)$ respectively, belong to this case. The second case is when $(-1)^s p\,\mathrm{Det}O_\sigma = -1$ and $O_\sigma \neq \mathbb{1}$. In this case, $E_{n,B}(O_\sigma\mathbf{k}) = -E_{n,B}(\mathbf{k})$ and this implies that the minimum and maximum values of LLS has the same magnitude but opposite in sign, i.e., $\max E_{n,B}(\mathbf{k}) = -\min E_{n,B}(\mathbf{k})$. Notable examples are time-reversal symmetry T and reflection symmetry R.

Symmetry constraint on $\chi^{nm}_{xy}(\mathbf{k})$

Although the symmetry analysis for the modified band dispersion $E_{n,B}(\mathbf{k})$ is sufficient for studying the Landau level spreading (LLS), the symmetry analysis for $\chi^{nm}_{xy}(\mathbf{k})$ provides some useful insights for understanding of the LLS as the inter-band coupling. From Eqs. (21) and (22), we obtain the relation between $\chi^{nm}_{ij}(\mathbf{k})$ and $\chi^{nm_\sigma}_{ij}(O_\sigma\mathbf{k})$ where the band m and the band m_σ are related by the symmetry σ so that $\varepsilon_m(\mathbf{k}) = p\,\varepsilon_{m_\sigma}(O_\sigma\mathbf{k})$:

$$\begin{aligned} \chi^{nm_\sigma}_{ij}(O_\sigma\mathbf{k}) =& \left[\langle\partial_{\mathbf{k}'_i} u_n(\mathbf{k}')|u_{m_\sigma}(\mathbf{k}')\rangle\langle u_{m_\sigma}(\mathbf{k}')|\partial_{\mathbf{k}'_j} u_n(\mathbf{k}')\rangle\right]_{\mathbf{k}'=O_\sigma\mathbf{k}} \\ =&[O_\sigma]_{ii'}[O_\sigma]_{jj'}\,\partial_{i'}\left(e^{i\phi_n(\mathbf{k})}\overline{\langle u_n(\mathbf{k})|}^s U_\sigma(\mathbf{k})^\dagger\right) \\ &\times\left(U_\sigma(\mathbf{k})\overline{|u_m(\mathbf{k})\rangle\langle u_m(\mathbf{k})|}^s U_\sigma(\mathbf{k})^\dagger\right)\partial_{j'}\left(U_\sigma(\mathbf{k})\overline{|u_n(\mathbf{k})\rangle}^s e^{-i\phi_n(\mathbf{k})}\right) \\ =&[O_\sigma]_{ii'}[O_\sigma]_{jj'}\overline{\chi^{nm}_{i'j'}(\mathbf{k})}^s. \end{aligned} \tag{26}$$

Let us now apply Eq. (26) to i) chiral symmetry C, ii) space-time-inversion symmetry I_{ST}, iii) a combined symmetry $C_{\mathrm{ST}} = C\circ I_{\mathrm{ST}}$ with C and I_{ST}, iv) time-reversal symmetry T, and v) reflection symmetry

i) Chiral symmetry C is characterized by $\mathfrak{g}_C = (O_\sigma, s, p) = (\mathbb{1}, 0, -1)$, and Eq. (26) implies $\chi^{nm_\sigma}_{ij}(\mathbf{k}) = \chi^{nm}_{ij}(\mathbf{k})$. Noting that $\varepsilon_m(\mathbf{k}) = -\varepsilon_{m_\sigma}(\mathbf{k})$, we obtain

$$\sum_{a=m,m_\sigma}\varepsilon_a(\mathbf{k})\chi^{na}_{ij}(\mathbf{k}) = 0. \tag{27}$$

Thus, for each chiral-symmetric pair $|u_m(\mathbf{k})\rangle$ and $|u_{m_\sigma}(\mathbf{k})\rangle$, the contribution to the modified band dispersion $E_{n,B}(\mathbf{k})$ cancels out.

ii) Space-time inversion I_{ST} define by $\mathfrak{g}_{I_{\mathrm{ST}}} = (\mathbb{1}, 1, 1)$ imposes the condition $\chi^{nm_\sigma}_{ij}(\mathbf{k}) = \left(\chi^{nm}_{ij}(\mathbf{k})\right)^*$. In I_{ST}-symmetric system, let us suppose non-degenerate band structure with band labeling $m = m_\sigma$ with $\varepsilon_m(\mathbf{k}) = \varepsilon_{m_\sigma}(\mathbf{k})$ for simplicity. Hence,

$$\varepsilon_m(\mathbf{k})\,\mathrm{Im}\,\chi^{nm}_{ij}(\mathbf{k}) = 0, \tag{28}$$

and contribution from each energy eigenstate $|u_m(\mathbf{k})\rangle$ vanishes.

iii) Let us consider $C_{\rm ST} = C \circ I_{\rm ST}$, a combination of chiral and space-time-inversion symmetries, characterized by $\mathfrak{g}_{C_{\rm ST}} = (\mathbb{1}, 1, -1)$. For this symmetry, the condition $\chi_{ij}^{nm_\sigma}(\mathbf{k}) = \left(\chi_{ij}^{nm}(\mathbf{k})\right)^*$ is imposed. This symmetry constraint is similar to $I_{\rm ST}$-symmetric system, but now $\varepsilon_m(\mathbf{k}) = -\varepsilon_{m_\sigma}(\mathbf{k})$. Hence,

$$\sum_{\{\varepsilon_a(\mathbf{k})>0\}} \varepsilon_a(\mathbf{k})\,\mathrm{Im}\,\chi_{ij}^{na}(\mathbf{k}) = \sum_{\{\varepsilon_{\bar{a}}(\mathbf{k})<0\}} \varepsilon_{\bar{a}}(\mathbf{k})\mathrm{Im}\,\chi_{ij}^{n\bar{a}}(\mathbf{k}). \tag{29}$$

In this system, both energy eigenstates with positive and negative energy eigenvalues contribute to the LLS with the same sign. In the main text, we discuss the spin-orbit-coupled (SOC) Lieb model. This model possesses $C_{\rm ST}$,

$$C_{\rm ST} H_{\rm socL}(\mathbf{k}) C_{\rm ST}^{-1} = -H_{\rm socL}(\mathbf{k}) \quad \text{where} \quad C_{\rm ST} = \mathrm{Diag}(1,-1,1)\mathcal{K}. \tag{30}$$

This symmetry leads to $\chi_{{\rm socL},xy}^{{\rm fb},+}(\mathbf{k}) = \left(\chi_{{\rm socL},xy}^{{\rm fb},-}(\mathbf{k})\right)^*$.

iv) Time-reversal symmetry T is characterized by $\mathfrak{g}_T = (-\mathbb{1}, 1, 1)$, and $\chi_{ij}^{nm_\sigma}(-\mathbf{k}) = \left(\chi_{ij}^{nm}(\mathbf{k})\right)^*$ consequently. Noting that $\varepsilon_m(\mathbf{k}) = \varepsilon_{m_\sigma}(-\mathbf{k})$, we obtain

$$\varepsilon_m(\mathbf{k})\,\mathrm{Im}\,\chi_{ij}^{nm}(\mathbf{k}) = -\varepsilon_{m_\sigma}(-\mathbf{k})\,\mathrm{Im}\,\chi_{ij}^{nm_\sigma}(-\mathbf{k}). \tag{31}$$

Thus, $E_{n,B}(\mathbf{k}) = -E_{n,B}(-\mathbf{k})$, and this implies $\max E_{n,B}(\mathbf{k}) = -\min E_{n,B}(\mathbf{k})$.

v) For a discussion on reflection symmetry R, we consider R_x, characterized by $\mathfrak{g}_{R_x} = (\mathrm{Diag}(-1,1), 0, 1)$, for convenience. R_x imposes $\chi_{ij}^{nm_\sigma}(-k_x, k_y) = -\chi_{ij}^{nm}(\mathbf{k})$. Considering $\varepsilon_m(\mathbf{k}) = \varepsilon_{m_\sigma}(-k_x, k_y)$, we obtain

$$\varepsilon_m(\mathbf{k})\,\mathrm{Im}\,\chi_{ij}^{nm}(\mathbf{k}) = -\varepsilon_{m_\sigma}(-k_x, k_y)\,\mathrm{Im}\,\chi_{ij}^{nm_\sigma}(-k_x, k_y), \tag{32}$$

and $E_{n,B}(\mathbf{k}) = -E_{n,B}(-k_x, k_y)$. In similar to iv), we conclude that $\max E_{n,B}(\mathbf{k}) = -\min E_{n,B}(\mathbf{k})$.

Let us comment on the case where some bands are degenerate. When the band m is degenerate with other band(s), $\chi_{ij}^{nm}(\mathbf{k})$ or $\varepsilon_m(\mathbf{k})\,\mathrm{Im}\,\chi_{ij}^{nm}(\mathbf{k})$ alone is not gauge invariant. Let us suppose that the D number of bands, whose band indices are $m_1, m_2, \dots, m_D$, form a set of degenerate bands $\{m\}$, i.e., $(m_1, m_2, \dots, m_D) \in \{m\}$. Then, $\sum_{a\in\{m\}} \chi_{ij}^{na}(\mathbf{k})$ and $\sum_{a\in\{m\}} \varepsilon_a(\mathbf{k})\,\mathrm{Im}\,\chi_{ij}^{na}(\mathbf{k})$ are gauge invariant under the gauge transformation,

$$|u_{m_i}(\mathbf{k})\rangle \to |u_{m_j}(\mathbf{k})\rangle\, G_{m_j m_i}(\mathbf{k}) \quad (i,j = 1,2,\dots,D), \tag{33}$$

where $G_{m_j m_i}(\mathbf{k})$ is D-by-D unitary matrix.

Supplementary Note 3. Landau level spreading of flat-band system with chiral symmetry

In this Supplementary Note, we discuss the LLS of flat-band system in the presence of chiral symmetry. As discussed in the main text and Supplementary Note 1, the LLS of chiral-symmetric system is forbidden in finite or all range of magnetic flux. First, let us discuss some properties of chiral-symmetric system in the zero magnetic flux. For chiral-symmetric Hamiltonian, the symmetry relation,

$$CH(\mathbf{k})C^{-1} = -H(\mathbf{k}) \tag{34}$$

holds. The implies that eigenstates $|u_a(\mathbf{k})\rangle$ and $|u_{\bar{a}}(\mathbf{k})\rangle$, having energy eigenvalues $\varepsilon_a(\mathbf{k})$ and $\varepsilon_{\bar{a}}(\mathbf{k}) = -\varepsilon_a(\mathbf{k})$ respectively, are related by the chiral symmetry operator C:

$$C|u_a(\mathbf{k})\rangle = |u_{\bar{a}}(\mathbf{k})\rangle B(\mathbf{k})_{\bar{a}a}. \tag{35}$$

Here, $B(\mathbf{k})$ denotes the sewing matrix for the chiral symmetry. The chiral eigenvalues c are given by the eigenvalues of $B_{\bar{a}a}(\mathbf{k})$. When $\varepsilon_{\bar{a}}(\mathbf{k}) = -\varepsilon_a(\mathbf{k}) \neq 0$, the sewing matrix is equal to

$$B(\mathbf{k})_{\bar{a}a} = \begin{pmatrix} 0 & 1 \\ 1 & 0 \end{pmatrix}, \tag{36}$$

up to unitary transformation, and its eigenvalues are given by $c = \pm 1$. Conversely, this implies that two degenerate flat-bands with zero energy having opposite chiral eigenvalues $c = \pm 1$ can be gapped under chiral-symmetric perturbations. However, a set of zero-energy flat bands cannot be gapped when all the chiral eigenvalues of such flat bands are equal. Since the sewing

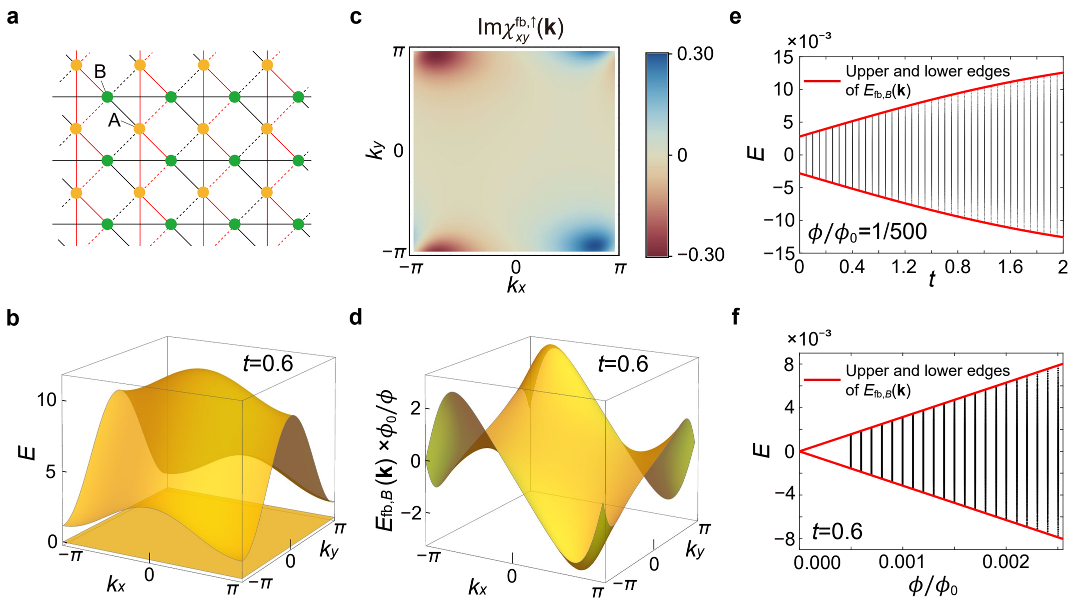

Supplementary Figure 1. **Landau level spreading of a flat-band system with time-reversal symmetry.** **a** Lattice structure for the T-symmetric checkerboard model. Neighboring sites connected by the same type of bonds (a solid or dotted line) with the same color have the same hopping amplitudes. **b** The band structure of $H_T(\mathbf{k})$. **c** Distribution of $\mathrm{Im}\chi_{xy}^{\mathrm{fb},\uparrow}(\mathbf{k})$. **d** The modified band dispersion $E_{\mathrm{fb},B}(\mathbf{k})$ of the flat band in the presence of magnetic flux. **e** Landau level spectra of the flat band (black dots) as a function of t for magnetic flux $\phi/\phi_0 = 1/500$. **f** Landau level spectra of the flat band (black dots) as a function of magnetic flux ϕ/ϕ_0 for $t = 0.6$. **e, f** The upper and lower bounds of Landau levels are equal in magnitude but opposite in sign.

matrix $B(\mathbf{k})_{\bar{a}a}$ for all bands is identical to the chiral symmetry operator C up to unitary transformation, the minimum number of zero-energy flat bands, guaranteed to be exists due to the chiral symmetry, is given by $|\mathrm{Tr}[C]|$.

In the magnetic finite flux, the system still remains chiral symmetric. This is because chiral symmetry arises from a specific way of choosing the hoppings between the sublattices, and it is not changed by the Peierls substitution. For the magnetic unit cell q times larger than the original unit cell, the minimal number of flat bands are equal to $q|\mathrm{Tr}[C]|$ where q is determined by the magnetic flux $\phi/\phi_0 = q_0\frac{p}{q}$ ($q_0, p, q \in \mathbb{Z}$). Note that q_0 is a constant dependent on the gauge choice and the models. These zero-energy flat bands still have the same chiral eigenvalues, thus these are protected by chiral symmetry ($C_{\mathrm{new}} = \oplus_{i=1}^{q} C$ now). Hence, unless a gap between zero-energy and non-zero-energy Landau levels closes as the magnetic flux increases, the LLS is forbidden even in the finite magnetic flux when the number of flat bands is $q|\mathrm{Tr}[C]|$ at the zero magnetic flux. When such a gap closing occurs at $\phi/\phi_0 = \varphi_*$, the LLS can be finite for the magnetic flux larger than φ_*. Nevertheless, there must be at least the $q|\mathrm{Tr}[C]|$ number of zero-energy Landau levels.

Supplementary Note 4. More lattice models

Time-reversal-symmetric system on the checkerboard lattice

When a time-reversal symmetry T exists at zero magnetic field, the LLS of an IFS has the minimum and maximum values of the same magnitude but opposite in sign. We consider a T-symmetric system defined in the checkerboard lattice. The lattice structure is shown in Supplementary Fig. 1a. The on-site potentials at A- and B-sites are set to be 5 and $1+t^2$, respectively. The hopping parameters are set to 2 along the red solid line, t along the black sold line, 1 along the red dashed lines, and $2t$ along the black dashed lines. The tight-binding Hamiltonian is written as

$$H_T(\mathbf{k}) = \begin{pmatrix} 5 + 4\cos k_y & 2te^{-i\mathbf{k}\cdot\mathbf{d}_1} + 2e^{-i\mathbf{k}\cdot\mathbf{d}_2} + e^{i\mathbf{k}\cdot\mathbf{d}_1} + te^{i\mathbf{k}\cdot\mathbf{d}_2} \\ 2te^{i\mathbf{k}\cdot\mathbf{d}_1} + 2e^{i\mathbf{k}\cdot\mathbf{d}_2} + e^{-i\mathbf{k}\cdot\mathbf{d}_1} + te^{-i\mathbf{k}\cdot\mathbf{d}_2} & 1 + t^2 + 2t\cos k_x \end{pmatrix}, \quad (37)$$

where $\mathbf{d}_1 = (1/2, 1/2)$ and $\mathbf{d}_2 = (-1/2, 1/2)$. This Hamiltonian gives a zero-energy flat band, and a dispersive band with energy $\varepsilon_{T,\uparrow}(\mathbf{k}) = 6 + t^2 + 2t\cos k_x + 4\cos k_y > 0$ as show in Supplementary Fig. 1b. The time-reversal symmetry operator is given by the complex conjugation $\mathcal{K}$, i.e., $T = \mathcal{K}$, which gives a symmetry relation,

$$TH_T(\mathbf{k})T^{-1} = H_T(-\mathbf{k}). \quad (38)$$

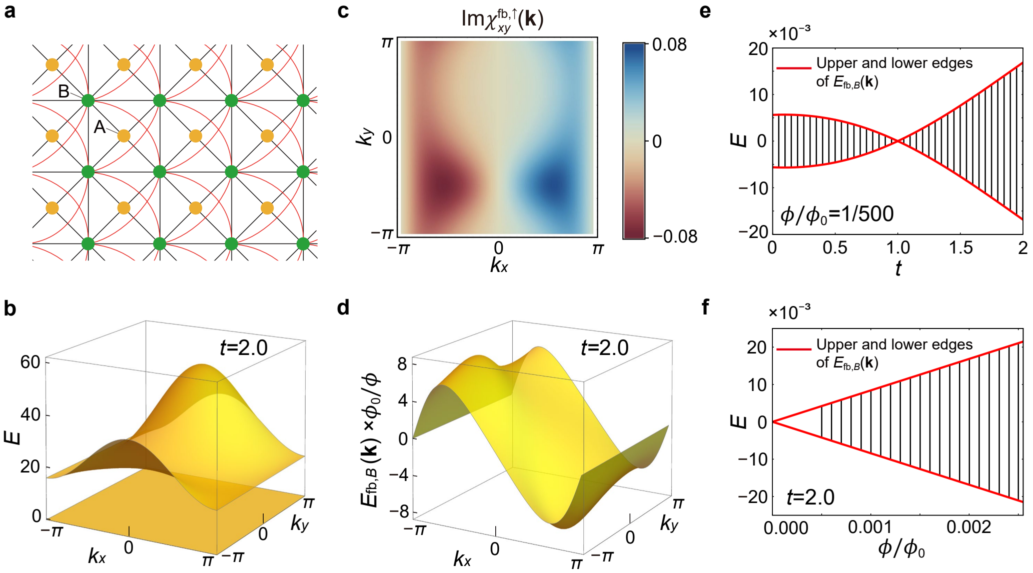

Supplementary Figure 2. **Landau level spreading of a flat-band system with reflection symmetry.** **a** The lattice structure for the I_{ST}-symmetric checkerboard model. The red and black lines denote the hopping processes between A and B sublattices. **b** The band structure of $H_R(\mathbf{k})$ with $t = 2.0$. **c** Distribution of $\mathrm{Im}\chi_{xy}^{\mathrm{fb},\uparrow}(\mathbf{k})$ with $t = 2.0$. **d** The modified band dispersion $E_{\mathrm{fb},B}(\mathbf{k})$ of the flat band in the presence of magnetic flux. **e** Landau level spectra of the flat band (black dots) as a function of t for magnetic flux $\phi/\phi_0 = 1/500$. We note that space-time inversion symmetry I_{ST} exist when $t = 1.0$, thus the LLS is strongly suppressed. **f** Landau level spectra of the flat band (black dots) as a function of magnetic flux ϕ/ϕ_0 for $t = 2.0$. **e**, **f** The upper and lower bounds of Landau levels are equal in magnitude but opposite in sign.

The analytic form of the fidelity tensor $\chi_{xy}^{nm}(\mathbf{k})$ is given by

$$\chi_{xy}^{\mathrm{fb},\uparrow}(\mathbf{k}) = \frac{(3 + 4i\sin k_y)(t^2 + 2i\sin k_x - 1)}{4\left(\varepsilon_{T,\uparrow}(\mathbf{k})\right)^2}. \tag{39}$$

Then, the modified band dispersion for the flat band is given by

$$E_{\mathrm{fb},B}(\mathbf{k}) = -\frac{\pi(3t\sin k_x + 2(t^2 - 1)\sin k_y)}{\varepsilon_{T,\uparrow}(\mathbf{k})}\frac{\phi}{\phi_0}. \tag{40}$$

In Supplementary Fig. 1c, d, $\mathrm{Im}\,\chi_{xy}^{\mathrm{fb},\uparrow}(\mathbf{k})$ and $E_{\mathrm{fb},B}(\mathbf{k})$ are shown. Note that $E_{\mathrm{fb},B}(-\mathbf{k}) = -E_{\mathrm{fb},B}(\mathbf{k})$. Consequently, the Landau levels of the flat band spread in both positive and negative energy directions with the same amounts as shown in Supplementary Fig. 1e, f.

Reflection-symmetric system on the checkerboard lattice

In similar to time-reversal symmetric system at the zero magnetic field, we expect that the LLS whose the minimum and maximum values have the same magnitude but opposite in sign, for reflection R symmetric system at the zero field. We consider a R-symmetric system defined in the checkerboard lattice. This model is symmetric under reflection symmetry R in the x-direction. The lattice structure is shown in Supplementary Fig. 2a. The tight-binding Hamiltonian consists of the hopping processes up to the next-to-next neighbor hopping. In momentum space, the Hamiltonian is written as

$$H_R(\mathbf{k}) = |\phi(\mathbf{k})\rangle\langle\phi(\mathbf{k})|, \tag{41}$$

$$|\phi(\mathbf{k})\rangle = (ie^{-i\mathbf{k}\cdot\mathbf{d}_1} + ie^{-i\mathbf{k}\cdot\mathbf{d}_2} + te^{i\mathbf{k}\cdot\mathbf{d}_1} + te^{i\mathbf{k}\cdot\mathbf{d}_2}, 4)^T, \tag{42}$$

where $\mathbf{d}_1 = (1/2, 1/2)$ and $\mathbf{d}_2 = (-1/2, 1/2)$. The band structure for $t = 2.0$ is shown in Supplementary Fig. 2b. The flat band's energy is zero and the energies of dispersive band is

$$\varepsilon_{R,\uparrow}(\mathbf{k}) = 18 + 2t^2 + 2(1 + t^2)\cos k_x + 4t\sin k_y + 2t\sin(k_x + k_y) - 2t\sin(k_x - k_y). \tag{43}$$

The reflection symmetry operator is given by $R = \mathrm{Diag}(1, 1)$ which gives a symmetry relation,

$$RH_R(\mathbf{k})R^{-1} = H_R(-k_x, k_y). \tag{44}$$

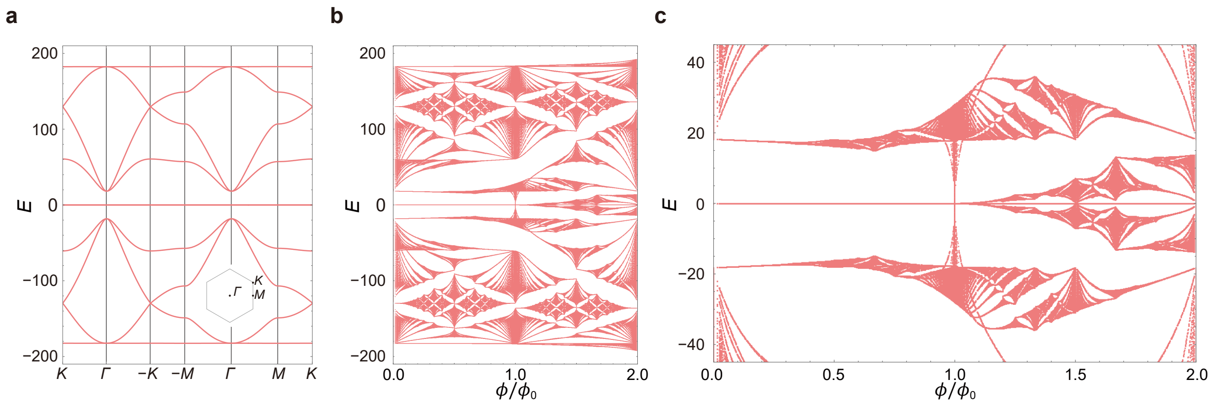

Supplementary Figure 3. **Landau level spreading of 10-band TBG model.** **a** The band structure for 10-band TBG model proposed in Ref. [3]. The Brillouin zone and the high-symmetry points are defined in the inset. **b** The Hofstadter spectrum exhibits the zero LLS of flat band at the magnetic flux ϕ/ϕ_0 in a range of $0 \leq \phi/\phi_0 < 1$. When $\phi/\phi_0 = 1$, a gap closing at $E = 0$ occurs, then the finite LLS is observed for $\phi/\phi_0 > 1$. **c** A zoom-in of the Hofstadter spectrum shown in **b**. Chiral symmetry guarantees the zero-energy Landau levels despite the appearance of non-zero LLS.

The analytic form of the fidelity tensor $\chi_{xy}^{nm}(\mathbf{k})$ is given by

$$\chi_{xy}^{\text{fb},\uparrow}(\mathbf{k}) = \frac{8i\sin k_x(t^2 + 2i\cos k_y - 1)}{(\varepsilon_{R,\uparrow}(\mathbf{k}))^2}. \tag{45}$$

Then, the modified band dispersion for the flat band is given by

$$E_{\text{fb},B}(\mathbf{k}) = -\frac{16\pi(t^2-1)\sin k_x}{\varepsilon_{R,\uparrow}(\mathbf{k})}\frac{\phi}{\phi_0}. \tag{46}$$

In Supplementary Fig. 2c, d, $\text{Im}\,\chi_{xy}^{\text{fb},\uparrow}(\mathbf{k})$ and $E_{\text{fb},B}(\mathbf{k})$ are shown. Note that $E_{\text{fb},B}(-k_x, k_y) = -E_{\text{fb},B}(\mathbf{k})$. Consequently, the Landau levels of the flat band spread in both positive and negative energy directions with the same amounts as shown in Supplementary Fig. 2e, f.

Ten-band model for twisted bilayer graphene

In this Supplementary Note, we discuss one more example for chiral-symmetric system. For this, we consider the ten-band model for twisted-bilayer graphene (TBG) proposed in Ref. 3. The band structure and the Hofstadter spectrum is shown in Supplementary Fig. 3a and 3b, c, respectively. The band structure exhibits two degenerate flat bands at zero energy when the on-site potential is neglected. Moreover, these flat bands have fragile topology protected by $C_{2z} \circ T$ [3–6]. Although this system is not our interest in this paper as we focus only on an isolated and topologically trivial flat band, we study this system in detail as an important example of chiral-symmetric flat-band system.

As shown in Supplementary Fig. 3b, c, There is no LLS in the Hofstadter spectrum for $0 \leq \phi/\phi_0 < 1$. This is because the chiral symmetry operator of this system is given by

$$C = \text{Diag}(-1,-1,-1,-1,1,1,1,1,1,1), \tag{47}$$

and $\text{Tr}[C] \neq 0$ at the zero magnetic field. According to the discussion in Supplementary Note 2, this implies the zero LLS in a finite range of the magnetic flux. Interestingly, the gap near $E = 0$ closes at $\phi/\phi_0 = 1$, and then the LLS is developed after the gap closing, as shown in Supplementary Fig. 3c. The connection between the gap closing and fragile topology is discussed in Ref. 7.

Supplementary References

[1] Chang, M.-C. & Niu, Q. Berry phase, hyperorbits, and the Hofstadter spectrum: Semiclassical dynamics in magnetic Bloch bands. *Physical Review B* **53**, 7010 (1996).

[2] Sundaram, G. & Niu, Q. Wave-packet dynamics in slowly perturbed crystals: Gradient corrections and Berry-phase effects. *Physical Review B* **59**, 14915 (1999).

[3] Po, H. C., Zou, L., Senthil, T. & Vishwanath, A. Faithful tight-binding models and fragile topology of magic-angle bilayer graphene. *Physical Review B* **99**, 195455 (2019).
[4] Po, H. C., Watanabe, H. & Vishwanath, A. Fragile topology and Wannier obstructions. *Physical Review Letters* **121**, 126402 (2018).
[5] Ahn, J., Park, S. & Yang, B.-J. Failure of Nielsen-Ninomiya theorem and fragile topology in two-dimensional systems with space-time inversion symmetry: application to twisted bilayer graphene at magic angle. *Physical Review X* **9**, 021013 (2019).
[6] Song, Z. *et al.* All magic angles in twisted bilayer graphene are topological. *Physical Review Letters* **123**, 036401 (2019).
[7] Lian, B., Xie, F. & Bernevig, B. A. Landau level of fragile topology. *Physical Review B* **102**, 041402(R) (2020).
